# Decentralized Control Mechanism for Determination of Moving Direction in Brittle Stars With Penta-Radially Symmetric Body

**DOI:** 10.3389/fnbot.2019.00066

**Published:** 2019-08-23

**Authors:** Takeshi Kano, Daichi Kanauchi, Hitoshi Aonuma, Elizabeth G. Clark, Akio Ishiguro

**Affiliations:** ^1^Research Institute of Electrical Communication, Tohoku University, Sendai, Japan; ^2^Research Center of Mathematics for Social Creativity, Research Institute for Electronic Science, Hokkaido University, Sapporo, Japan; ^3^Department of Geology and Geophysics, Yale University, New Haven, CT, United States

**Keywords:** brittle star, decentralized control, autonomous robot, locomotion, determination of moving direction

## Abstract

A brittle star, an echinoderm with penta-radially symmetric body, can make decisions about its moving direction and move adapting to various circumstances despite lacking a central nervous system and instead possessing a rather simple distributed nervous system. In this study, we aimed to elucidate the essential control mechanism underlying the determination of moving direction in brittle stars. Based on behavioral findings on brittle stars whose nervous systems were lesioned in various ways, we propose a phenomenological mathematical model. We demonstrate via simulations that the proposed model can well reproduce the behavioral findings. Our findings not only provide insights into the mechanism for the determination of moving direction in brittle stars, but also help understand the essential mechanism underlying autonomous behaviors of animals. Moreover, they will pave the way for developing fully autonomous robots that can make decisions by themselves and move adaptively under various circumstances.

## 1. Introduction

Most robots are designed to perform given tasks in predefined environments and lack the ability to autonomously determine and move toward their moving direction while adapting to various unpredictable situations. In contrast, animals can feasibly adapt to the unpredictable real-world and move toward their desired direction. Interestingly, this ability is not unique to higher organisms with sophisticated brains, but is inherent even in primitive living organisms (Meyer et al., 2017). This fact suggests that decisions are not made solely by a central controller, i.e., brain, and that decentralized control plays a significant role in animal locomotion. Many studies have been devoted to elucidate the decentralized control mechanism underlying animals' adaptive locomotion (Kimura et al., 2007; Schilling et al., 2013; Kano et al., 2017a,c), yet they have not succeeded in achieving both autonomous determination of moving direction and adaptation to unpredictable circumstances simultaneously. Clarifying it will help understand the mechanism of autonomous behaviors of animals as well as develop fully autonomous robots that can make decisions by themselves and behave adaptively and reasonably on this basis.

**Figure 1:**
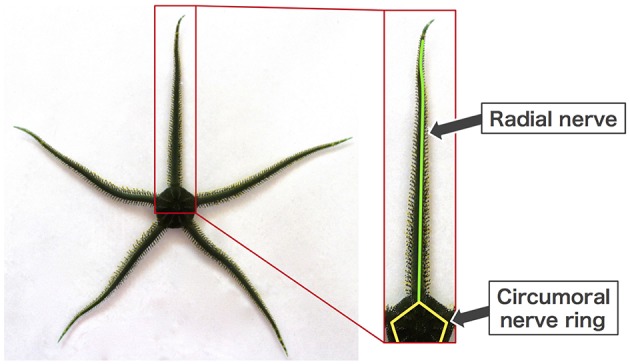
Body and nerve structure of a brittle star (*Ophiarachna incrassata*).

Brittle stars, a group of echinoderms that locomote on the sea floor, are a suitable model for addressing the above-mentioned issue. They have a penta-radially symmetric body in which five arms radiate from the central disc and are capable of moving omni-directionally (Figure 1) (Arshavskii et al., 1976b; Astley, 2012; Kano et al., 2012; Watanabe et al., 2012). They lack a central nervous system but instead possess a rather simple distributed nervous system consisting of radial nerves along the arms, which join a circumoral nerve ring at the disc (Figure 1) (Cobb and Stubbs, 1981). Despite such a simple nervous system, they can make decisions about their own movement (e.g., to escape from predators or to approach food), and once the decision is made, they locomote by coordinating their arm movements in real time (Arshavskii et al., 1976a,b; Astley, 2012; Matsuzaka et al., 2017; Clark et al., 2019). Moreover, they have outstanding resilience to bodily damage. Even after arbitrary loss of their arms, they promptly determine their moving direction and reorganize the coordination of the remaining arms to resume locomotion (Arshavskii et al., 1976a; Kano et al., 2017b).

It is likely that the moving direction is determined in the circumoral nerve ring by integrating sensory information detected at the arms. However, previous works on the locomotion of brittle stars (Arshavskii et al., 1976a,b; Astley, 2012; Kano et al., 2012, 2017b; Watanabe et al., 2012) did not elucidate the essential mechanism for the determination of moving direction. Recently, we have investigated the role of the circumoral nerve ring in the determination of moving direction through behavioral experiments (Clark et al., 2019). In particular, we observed the locomotion of brittle stars whose circumoral nerve ring was transected at various points. We found that brittle stars tended to move toward the direction opposite to the transected points, and that arms do not coordinate when the neural connection between the arms is lost by the transection. It is expected that these findings impart important insights into the determination of moving direction.

In this study, we propose a mathematical model that explains the behavioral findings on the nerve ring transection to deepen the understanding of the mechanism for the determination of moving direction in brittle star locomotion. In particular, based on our previous model for the adaptive inter-arm coordination of trimmed-arm brittle stars (Kano et al., 2017b), the extended model is proposed wherein the moving direction is modeled phenomenologically with an analogy of water tanks connected by tubes. We demonstrate via simulations that the proposed model well reproduces the results of the behavioral experiments.

## 2. Behavioral Findings

**Figure 2:**
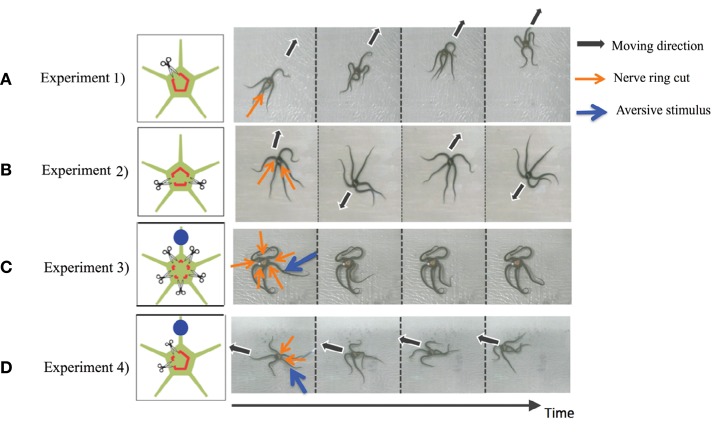
Results of the behavioral experiments: **(A)** Experiment 1, **(B)** Experiment 2, **(C)** Experiment 3, and **(D)** Experiment 4. Points where the nerve ring is cut and points where an aversive stimulus is added are indicated by scissors and blue circles, respectively. In the snapshots, the moving direction, points where the nerve ring is cut, and points where an aversive stimulus is added are indicated by black, orange, and blue arrows, respectively. Except for **(B)**, data was taken from the work by Clark et al. (2019).

In this section, we present representative results of the behavioral experiments in which the circumoral nerve ring was transected in various ways (Clark et al., 2019). In particular, here we present the following four cases (Figure 2):

- Experiment 1. The nerve ring was cut in one place.
- Experiment 2. The nerve ring was cut in two places, with each arm retaining at least one nerve ring connection with an adjacent arm.
- Experiment 3. The nerve ring connection between each arm was cut (i.e., five separations). Moreover, an aversive stimulus was added to one of the arms.
- Experiment 4. The nerve ring was cut in two places but on both sides of the same arm. Moreover, an aversive stimulus was added to one of the neurally connected arms.

Note that in all experiments, we first observed the behavior of intact brittle stars for 10 min. Then, we cut the nerve rings and observed the behavior for a further 10 min. We used potassium chloride (KCL) solution as the aversive stimuli. Detailed procedures and the results of the experiments are provided in the work by Clark et al. (2019).

The results are shown in Figure 2. In Experiment 1, brittle stars tended to move in the direction opposite to the point where the nerve ring was cut (Figure 2A and Supplementary Movie 1). In Experiment 2, the arms connected by the nerve ring tended to coordinate; however, neurally disconnected arms did not tend to coordinate. Namely, it was often observed that the two connected arms coordinated to move in a certain direction, whereas the other three arms coordinated to move in the opposite direction (Figure 2B and Supplementary Movie 2). In Experiment 3, the arms did not coordinate and, thus, locomotion was not observed. When a stimulus was applied to one of the arms, only the stimulated arm responded whereas the other arms did not respond (Figure 2C and Supplementary Movie 3). In Experiment 4, the four neurally connected arms coordinated to move away from the stimulus, whereas the neurally isolated arm did not coordinate with the other arms and, thus, did not contribute to locomotion (Figure 2D and Supplementary Movie 4).

In summary, the following findings were obtained from the above experiments.

1. Brittle stars tend to move to a direction opposite to the point where the nerve ring was cut.
2. Neurally isolated arms cannot coordinate with other arms.
3. Sensory input (i.e., stimulus) is transmitted to neurally connected arms and enables them to coordinate.

Thus, it is likely that neural connection in the nerve ring plays an important role in the determination of moving direction.

## 3. Model

Based on the above findings, we propose a mathematical model. We have previously proposed a decentralized control model for the inter-arm coordination and succeeded in reproducing the locomotion patterns of brittle stars in which several arms were amputated (Kano et al., 2017b). This model was implemented in a brittle star-like robot, and it adapted to physical damages like real brittle stars; thus, it likely captures the essence of brittle star locomotion. However, the moving direction is determined by the central command. Thus, we here modify our previous model so that the moving direction is determined in a self-organized manner at the nerve ring. We review our previous model in section 3.1, and then we propose the modified model in section 3.2.

### 3.1. Review of Our Previous Work

**Figure 3:**
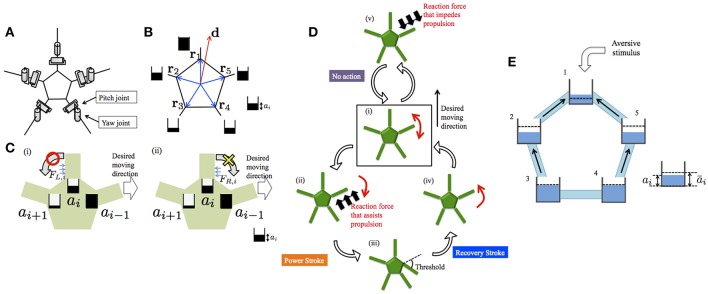
Schematics of the previous model (Kano et al., 2017b) and the proposed model. **(A)** Body system. **(B)** Definitions of **d** and **r**_*i*_. The *a*_*i*_ values are also shown schematically. **(C)** Evaluation of reaction force from the environment when *a*_*i*−1_ > *a*_*i*_ > *a*_*i*+1_. **(D)** Outline of the decentralized control mechanism. Detailed explanations are provided in the main text. **(E)** Schematic of the proposed model. Five water tanks are connected by tubes. Water level denotes *a*_*i*_. Dashed lines denote *ā*_*i*_. Black arrows indicate water flow. Schematics in **(A,C,D)** were reproduced from Kano et al. (2017b).

The schematic of the body system in our previous model (Kano et al., 2017b) is shown in Figure 3A. The body consists of a pentagonal central disc and five arms radiating from its vertices. Each arm has only two degrees of freedom, i.e., yaw and pitch joints. Each arm can detect the ground reaction force parallel to the ground. The reaction forces acting on the right- and left-hand sides of the *i*th arm are denoted by *F*_*R, i*_ and *F*_*L, i*_, respectively.

It is assumed that the desired moving direction of the center of the body is predetermined and is denoted by the vector **d**. On this basis, parameters *a*_*i*_ (*i* = 1, 2, 3, 4, 5) are defined as

(1) $$\begin{array}{r} a_{i}={\text{r}}_{i}\cdot \text{d}, \end{array}$$

where **r**_*i*_ denotes the vector pointing the proximal end of the *i*th arm from the viewpoint of the center of the disc. Thus, *a*_*i*_ is large when the proximal end of the *i*th arm is oriented toward the direction of motion from the viewpoint of the center of the central disc (Figure 3B).

Using the *a*_*i*_ values, each arm can evaluate whether the detected reaction force assists with propulsion toward the desired moving direction or not. In particular, *U*_*R, i*_ and *U*_*L, i*_, which are defined as

(2) $$\begin{array}{rcl} U_{R,i} & = & \text{max}\left[a_{i}-a_{i-1},0\right]F_{R,i},\\ U_{L,i} & = & \text{max}\left[a_{i}-a_{i+1},0\right]F_{L,i}, \end{array}$$

represent to what extent the detected reaction force from the right/left assists with propulsion toward the desired moving direction, and the *i*th arm can make the evaluation on the basis of the values of *U*_*R, i*_ and *U*_*L, i*_. For example, let us consider the case in which the *i*th arm is oriented toward the moving direction, i.e., *a*_*i*−1_ > *a*_*i*_ > *a*_*i*+1_ (Figure 3C). When the *i*th arm experiences a reaction force from the left, *U*_*L, i*_ is positive and, thus, the reaction force assists with propulsion. Meanwhile, when the *i*th arm receives a reaction force from the right, *U*_*R, i*_ is zero and, thus, the reaction force does not assist with propulsion.

Based on the above, the torque generated at each joint is determined according to the following rule (Figure 3D). First, each arm moves randomly to detect the reaction force against the ground (Figure 3Di). If the reaction force assists with propulsion toward the desired moving direction, the arm pushes against the ground, i.e., a power stroke begins (Figure 3Dii). Then, a recovery stroke begins when the joint angle reaches a certain threshold (Figures 3Diii,iv). On the other hand, if the reaction force impedes propulsion toward the desired moving direction, no action is generated (Figure 3Dv). Detailed mathematical formulas are provided in the Appendix and Kano et al. (2017b)

### 3.2. Proposed Model

In the above-mentioned model, *a*_*i*_ is determined by using the predetermined desired moving direction **d** (Equation 1). However, because real brittle stars likely determine their moving direction by integrating sensory information detected at each body part, it is natural to consider that *a*_*i*_ is determined in a self-organized manner. Hence, here we modify the model, focusing on how to control *a*_*i*_.

Unfortunately, a neurophysiological basis for the determination of *a*_*i*_ is lacking at the present stage. Hence, we model *a*_*i*_ phenomenologically: we control *a*_*i*_ by considering an analogy with water tanks connected with tubes (Figure 3E). In this analogy, five water tanks, each of which corresponds to each arm, are located on a plane and they are connected with tubes. The water level of the *i*th tank represents the value of *a*_*i*_. The water levels between adjacent tanks tend to decrease because water flows from a tank with higher level to that with lower level; thus, *a*_*i*_ evolves in a diffusive manner. In each tank, water is added or removed so that the water level approaches the target level. The target level increases and decreases when attractant and aversive stimuli are added to the corresponding arm, respectively.

Thus, the time evolution of *a*_*i*_ is described as follows:

(3) $$\begin{array}{r} \tau {\dot{a}}_{i}={\bar{a}}_{i}-a_{i}+D\left(a_{i-1}+a_{i+1}-2a_{i}\right), \end{array}$$

where τ is a time constant and *D* denotes the diffusion coefficient, which is related to the diameter of the tubes in Figure 3E. The target water level *ā*_*i*_ is given by

(4) $$\begin{array}{r} {\bar{a}}_{i}=c_{0}+s_{i}, \end{array}$$

where *c*_0_ is a positive constant and *s*_*i*_ denotes the stimulus applied to the *i*th arm, which is positive and negative in the case of attractant and aversive stimulus, respectively.

It is assumed in our model that a nerve ring cut corresponds to a tube cut and that water outflows from the cross-section of the cut tube. Thus, in the case of a nerve ring cut at the place between the *i*th and (*i*+1)th arms, for example, the time evolution of *a*_*i*_ is calculated by replacing *a*_*i*+1_ on the right-hand side of Equation (3) with zero.

We note that the definitions of *U*_*R,i*_ and *U*_*L,i*_ are slightly changed from Equation (2) as follows:

(5) $$\begin{array}{rcl} U_{R,i} & = & \text{tanh}\left\{\kappa_{u}\left(\text{max}\left[a_{i}-a_{i-1},0\right]F_{R,i}\right)\right\},\\ U_{L,i} & = & \text{tanh}\left\{\kappa_{u}\left(\text{max}\left[a_{i}-a_{i+1},0\right]F_{L,i}\right)\right\}. \end{array}$$

The hyperbolic tangent functions are introduced to make the parameter tuning feasible and to well mimic the behavioral findings, yet this change is not essential for the determination of moving direction.

## 4. Simulation

To validate the proposed model, we performed simulation experiments. Experimental conditions the same as Experiments 1–4 in section 2 were examined. The parameter values, which were determined by trial-and-error, are as follows: τ = 0.2 s, *c*_0_ = 1.0, *D* = 1.0 × 10^3^, λ = 2.0 s^−1^, β_yaw_ = 2.0, β_pitch_ = 30.0, γ_yaw_ = 5.0, γ_pitch_ = 10.0, *κ*_u_ = 1.0 × 10^2^, γ_*s*_ = 80.0, θ_*th*_ = π/6 rad. The proportional gains of the yaw and joints are 1.5 and 0.7 kgs^−2^, respectively, while the derivative gains of the yaw and pitch joints are 0.6 and 0.0 kgs^−1^, respectively.

**Figure 4:**
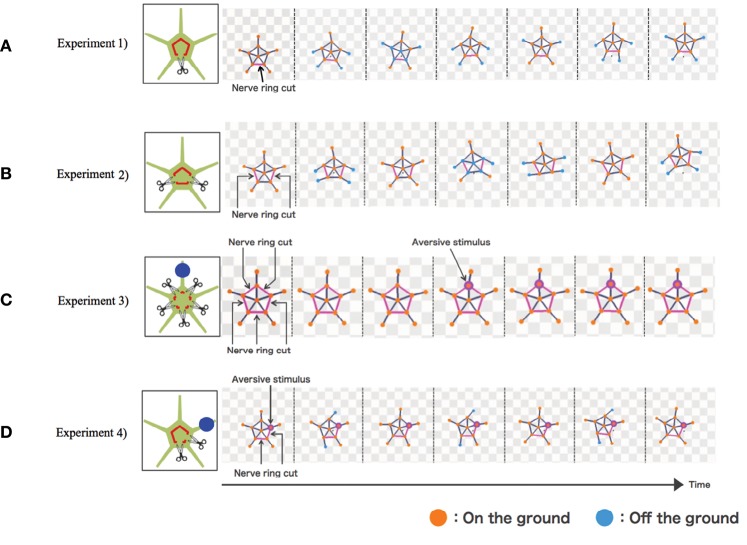
Simulation results: **(A)** Experiment 1, **(B)** Experiment 2, **(C)** Experiment 3, and **(D)** Experiment 4. Points where the nerve ring is cut and points where an aversive stimulus is added are indicated by scissors and blue circles, respectively. In the snapshots, points where the nerve ring is cut are indicated by pink lines. Points where aversive stimuli are added are indicated by pink circles. Mass points are colored orange and blue when they contact with and lift off the ground, respectively.

The results are shown in Figure 4. In Experiment 1, the simulated brittle star tended to move in the direction opposite to the point where the nerve ring was cut (Figure 4A and Supplementary Movie 5). In Experiment 2, the connected two arms coordinated to move in a certain direction, whereas the other three arms coordinated to move in the opposite direction (Figure 4B and Supplementary Movie 6). In Experiment 3, the arms did not coordinate and, thus, locomotion was not observed. Locomotion was not initiated even when a stimulus was applied to one of the arms (Figure 4C and Supplementary Movie 7). In Experiment 4, the four neurally connected arms coordinated to move away from the stimulus, whereas the neurally isolated arm did not coordinate with other arms and, thus, did not contribute to locomotion (Figure 4D and Supplementary Movie 8). Thus, the behaviors of real brittle stars shown in section 2 were generally well reproduced by the proposed model.

However, there still exists a difference between the simulated and real brittle stars. Each arm of the brittle star in Experiment 3 and the neurally isolated arm in Experiment 4 looked relaxed and did not actively push against the ground (Figures 2C,D). Meanwhile, in the simulation, these arms generate torques and actively pushed themselves against the ground (Figures 4C,D). This is because the terms max[*a*_*i*_ − *a*_*i*−1_] and max[*a*_*i*_ − *a*_*i*+1_] in Equation (5) are always positive for the neutrally isolated arm.

## 5. Conclusion and Future Work

In this study, we have focused on a brittle star which can autonomously determine the moving direction and move toward it adaptively under various circumstances by using its primitive distributed nervous system. Based on behavioral findings from the locomotion of brittle stars whose nerve rings were cut in various ways (Clark et al., 2019), we proposed a phenomenological mathematical model in which the direction of movement is determined in a self-organized manner, using an analogy with water tanks connected by tubes. We demonstrated via simulations that the behavioral findings can be well reproduced. Thus, our model likely captures the essence for the determination of moving direction in brittle stars.

We believe that this study imparts deep insights to biologists. While the previous models on animal locomotion (Kimura et al., 2007; Schilling et al., 2013; Kano et al., 2017a,c) focused on adaptation to various circumstances, the proposed model enables autonomous determination of the moving direction as well as adaptation. Thus, it will help understand the mechanism underlying autonomous behaviors of animals. Moreover, from an engineering perspective, the proposed control mechanism will be helpful in designing fully autonomous robots that can determine their moving direction as well as adapt to the circumstances encountered in real time, and it can potentially be used for disaster scenarios.

However, several problems still remain. First, a biological basis for the proposed model is still lacking. In particular, the physical meaning of *a*_*i*_ from a neurophysiological basis is unclear. Second, there still exists a discrepancy between the simulated and real brittle stars. Third, it is still unclear how the control mechanism of brittle star locomotion is related to that of other animals. Further neurophysiological studies and mathematical modeling on this basis are needed in the future. Extension of the proposed model to describe the coupling between the inter- and intra-arm coordination also remains as a future work.

## Data Availability

The raw data supporting the conclusions of this manuscript will be made available by the authors, without undue reservation, to any qualified researcher.

## Author Contributions

TK and AI: contributed the initial conception. TK, DK, HA, and EC: proposed the mathematical model. DK: performed simulations. TK: wrote the manuscript. DK, HA, EC, and AI: contributed to manuscript revision.

### Conflict of Interest Statement

The authors declare that the research was conducted in the absence of any commercial or financial relationships that could be construed as a potential conflict of interest.

## Supplementary Material

Supplementary Movie 1 The behavior of a brittle star in Experiment 1.

Supplementary Movie 2 The behavior of a brittle star in Experiment 2.

Supplementary Movie 3 The behavior of a brittle star in Experiment 3.

Supplementary Movie 4 The behavior of a brittle star in Experiment 4.

Supplementary Movie 5 Simulation result for Experiment 1.

Supplementary Movie 6 Simulation result for Experiment 2.

Supplementary Movie 7 Simulation result for Experiment 3.

Supplementary Movie 8 Simulation result for Experiment 4.

## Appendix

The joint torque is determined according to proportional-derivative control, and the time evolution of the target joint angles of the *i*th arm is defined as follows:

(A1) $$\begin{array}{r} {\dot{\Phi }}_{i}=\lambda \left(\Xi_{i}+{\text{P}}_{i}+{\text{R}}_{i}-\Phi_{i}\right), \end{array}$$

where λ is a positive constant and $\Phi_{i}={\left[\phi_{\text{yaw},i},\phi_{\text{pitch},i}\right]}^{\text{T}}$ with *ϕ*_yaw, *i*_ and *ϕ*_yaw, *i*_ being the target angles for the yaw and pitch joints, respectively. Note that the signs of the yaw and pitch joint angles are taken as positive when the arm bends leftward and upward with respect to the central disc, respectively. The vectors **Ξ**_*i*_, **P**_*i*_, and **R**_*i*_ contribute to the generation of the noise, power stroke, and recovery stroke, respectively. Hereafter, we describe mathematical formulas for these terms.

The noise vector **Ξ**_*i*_ is defined as

(A2) $$\begin{array}{r} \Xi_{i}=\left[\begin{array}{c} \alpha_{\text{yaw}}\xi_{\text{yaw},i}\\ \alpha_{\text{pitch}}\xi_{\text{pitch},i} \end{array}\right], \end{array}$$

where α_yaw_ and α_pitch_ are positive constants and ξ_yaw, *i*_ and ξ_pitch, *i*_ are uniform random numbers in the range [−1, 1], which vary with a fixed interval. It should be noted that the noise is essential because the robot cannot interact with the environment in the absence of the noise. Namely, if the noise is absent, the driving force of the robot does not exist, and the robot cannot detect the reaction force against the ground.

The power stroke vector, **P**_*i*_ is defined as

(A3) $$\begin{array}{r} {\text{P}}_{i}=\left[\begin{array}{c} -\beta_{\text{yaw}}\left(U_{R,i}-U_{L,i}\right)\\ -\beta_{\text{pitch}}\left(U_{R,i}+U_{L,i}\right) \end{array}\right], \end{array}$$

where β_yaw_ and β_pitch_ are positive constants, and *U*_*R,i*_ and *U*_*L,i*_ are defined in Equation (2) in the main text.

The recovery stroke vector **R**_*i*_ is defined as

(A4) $$\begin{array}{r} {\text{R}}_{i}=\left[\begin{array}{c} \gamma_{\text{yaw}}\left(S_{R,i}-S_{L,i}\right)\\ \gamma_{\text{pitch}}\left(S_{R,i}+S_{L,i}\right) \end{array}\right], \end{array}$$

where γ_yaw_ and γ_pitch_ are positive constants, and *S*_*R, i*_ and *S*_*L, i*_ are given by

(A5) $$\begin{array}{r} S_{R,i}=\frac{1}{2}\left[1+\text{tanh}\left\{\kappa_{s}\left(-\theta_{\text{yaw},i}-\theta_{th}\right)\right\}\right],\\ S_{L,i}=\frac{1}{2}\left[1+\text{tanh}\left\{\kappa_{s}\left(\theta_{\text{yaw},i}-\theta_{th}\right)\right\}\right], \end{array}$$

where κ_*s*_ is a positive constant, and θ_yaw, *i*_ is the yaw joint angle of the *i*th arm. When the joint angle reaches the threshold angle θ_*th*_ or −θ_*th*_, *S*_*L, i*_ or *S*_*R, i*_ increases, respectively; thus, the target joint angle changes, causing the *i*th arm to move in the opposite direction by lifting off from the ground.

In summary, the noise vector **Ξ**_*i*_ is essential for detecting the reaction force against the ground (Figure 3Di). The power stroke vector **P**_*i*_ is **0** unless the *i*th arm detects a reaction force that assists with propulsion; however, **P**_*i*_ acts in such a way that the *i*th arm can push against the ground when it detects an assistive reaction force (Figure 3Dii). The recovery stroke vector **R**_*i*_ is nearly equal to **0** unless the yaw joint angle of the *i*th arm exceeds the threshold ±θ_*th*_; however, **R**_*i*_ acts in such a way that the *i*th arm moves itself forward when the yaw joint angle reaches the threshold (Figures 3Diii,iv).
